# Entanglement generation by strong coupling between surface lattice resonance and exciton in an Al nanoarray-coated WS_2_ quantum emitter

**DOI:** 10.1186/s11671-023-03804-w

**Published:** 2023-03-06

**Authors:** Xiaoqi Shi, Zhihang Wang, Jiamin Xiao, Lingyao Li, Shibo Wei, Zhicheng Guo, Yi Wang, Wenxin Wang

**Affiliations:** 1 College of Physics and Optoelectronic Engineering, Harbin Engineering University, Harbin, 150001 China; 2 Qingdao Innovation and Development Base, Harbin Engineering University, Qingdao, 266500 China

**Keywords:** Strong coupling, Surface lattice resonance, Surface plasmon polarization, Entanglement

## Abstract

Strong light–matter interaction plays a central role in realizing quantum photonic technologies. The entanglement state, which results from the hybridization of excitons and cavity photons, forms the foundation of quantum information science. In this work, an entanglement state is achieved by manipulating the mode coupling between surface lattice resonance and quantum emitter into the strong coupling regime. At the same time, a Rabi splitting of 40 meV is observed. A full quantum model based on the Heisenberg picture is used to describe this unclassical phenomenon, and it perfectly explains the interaction and dissipation process. In addition, the observed concurrency degree of the entanglement state is 0.5, presenting the quantum nonlocality. This work effectively contributes to the understanding of nonclassical quantum effects arising from strong coupling and will intrigue more interesting potential applications in quantum optics.

## Introduction

Recently, the study of strong coupling between optical microcavities and quantum emitters has attracted great attention [1]. Strong coupling could manipulate the motion of a single photon, thus deriving applications such as all-optical switches [2], single-atom lasers [3] and quantum information processing [4]. When the coupling coefficient g between subsystems is larger than the loss rates of the cavity and quantum emitter, $\kappa$ and $\gamma$, respectively, i.e.$g^{2} > (\kappa^{2} + \gamma^{2} )/2$ [5], the interaction between light and matter reaches the strong coupling regime, which manifests the vacuum Rabi splitting in the spectra. The generated hybrid state can be defined as the entangled state [6], since it cannot be decomposed into the state products of its subsystem components. The degree of entanglement can be evaluated by concurrence and regulated by the coupling coefficient. As a superposition of quantum states, the entangled state [7] has attracted much attention due to its nonlocal coherence, which constitutes the basis of quantum cryptography and quantum teleportation [8]. Furthermore, the room-temperature strong coupling [9] promotes the generation of entangled states as well.

Since the strong coupling could reach the limit of quantum optics [10], it should be described by the cavity quantum electrodynamics (CQED) [11–13]. The coupling coefficient $g$ is proportional to the quality factor ($Q$) of the microcavity and is inversely proportional to the square root of the mode volume $V_{{\text{mode }}} (g \propto Q/\sqrt {V_{{{\text{mode}}}} } )$, $\kappa \propto 1/Q$, $Q = 2\pi \nu \frac{{E_{2} }}{{E_{1} }}$, $\nu$ is the resonance frequency of the cavity, *E*_2_ is the energy stored in the cavity and *E*_1_ is the stored energy lost per oscillation cycle [13].

Generally, $Q$ is directly related to the mode loss and describes the properties of the photon localization (lifetime). The *V*_mode_ of the optical cavity represents the localization ability of the optical mode in space, and a smaller *V*_mode_ presents higher optical field confinement and better photon localization in the nanostructure. Hence, both $Q$ and *V*_mode_ are important indicators to describe the cavity properties in CQED [13], and coherent resonances with high $Q$ and tiny *V*_mode_ are highly demanded.

Various optical microcavities are widely designed to increase the $Q$. For instance, the $Q$ of the whispering gallery mode [14] could reach $10^{6}$ while having a diameter of only a few tens of microns. In addition, various defects (such as point defects and line defects) are introduced into photonic crystals to confine the incident light and a photonic crystal microcavity with $Q$ of 10^5^–10^7^ [15] and optical *V*_mode_ of $\lambda^{3}$ is obtained, which can significantly enhance the coupling of photons and quantum emitters. Among these cavities, plasmonic structures have been extensively explored owing to their great features beyond the diffraction limit [16, 17].

Compared to conventional optical microcavities, the plasmonic structure has the ability of subwavelength optical field confinement [18], causing a sharp suppression in its *V*_mode_ as well as an enhanced and localized electric field, further increasing the value of $Q$/$\sqrt V$ [19]. For example, the plasmonic gold dimer [20] and silver nanorod [21] are used to construct microcavities. However, due to the constant collision of free electrons and atoms (ions) in metals, the surface plasmon mode has a high intrinsic loss and low $Q$ value, which limits further investigation and their potential applications. To address the issues above, a periodic plasmonic nanoarray [22] is proposed to provide a surface lattice resonance (SLR) [23] mode that can compensate for the loss by inducing scattering photons as a diffraction mode in the system.

The line width of SLR mode reduces remarkably and the $Q$ factor could reach 10^3^ [19] when compared with localized surface plasmon resonance (LSPR). This is because when a single particle expends to a periodical array, the radiative damping caused by the nanostructures could be offset by the scatting field. The diffraction orders (DOs) that interact with the LSPR will lead to the field’s redistribution that most of the electric fields concentrate on the edges of each structural unit. Since SLR retains the small *V*_mode_ of the surface plasmon and effectively reduces the radiative loss, it is attractive to be a subsystem in the strong coupling system and is expected to enhance the coupling coefficient $g$. On the other hand, $g = \mu \sqrt {\frac{{\pi \omega_{c} }}{h\varepsilon V}}$, the coupling coefficient $g$ is proportional to the dipole moment of the quantum emitter $\mu$. Therefore, the selection of quantum emitters is also critical for the generation of strong coupling. Generally, the transition metal dichalcogenides (TMDCs) have a large dipole moment [24], where the dipole moment of the single-layer WS_2_ is more prominent due to its single-layer direct band gap with high electron mobility, and it also possesses a long carrier lifetime and excellent nonlinear properties. As a result, WS_2_ is a great candidate to achieve strong coupling among quantum emitters. The material of metal plasmon is based on two points: It should be a relatively economical and manipulatable material, and should exhibit high plasmon density. From the band structure and optical properties, Al exhibits higher density of free electron, with three electrons per atom (Au and Ag has one electron per atom), and this could lead to a higher $\omega_{p}$ which represents the frequency range. The plasmon frequency is directly related to:$\omega_{p} = \sqrt {\frac{{ne^{2} }}{{\varepsilon_{0} m}}}$, where *n* is the density of free electrons, $\varepsilon_{0}$ is the dielectric permittivity of vacuum, e is the electric charge and m is the effective mass. For Al, the $\omega_{p } \approx 15\,{\text{eV}}$, but for Au and Ag, the $\omega_{p } \approx$ 8–9 eV. The relatively high value of $\omega_{p}$ indicates the frequency range of Al is broader [25]. Besides, Al is a relatively economical and manipulatable material that opens more avenues for fabrication and mass production. These requirements ensure the mass production and a big range of application. In this way, Al plasmon lattice is an excellent candidate for strong coupling.

This work proposes a composite system that consists of the plasmonic periodic nanoarray and a quantum emitter of WS_2_ film to obtain a strong coupling between the SLR and excitons. The generated strong coupling phenomenon with a Rabi splitting of 40 meV was investigated by using numerical simulation and theoretical calculation. A full quantum model was established to reveal the temporal dynamics of SLR–exciton entanglement. Concurrence [6], the nonlocal properties of the entanglement, and other nonclassical effects were described through the Wigner function distribution [26]. The simulation results fitted well with the full quantum model and theoretically proved the generation of strong coupling. Moreover, the evolution of the photon number over time suggested a periodic energy exchange between the two subsystems. It is believed that the results will have great significance for the realization of quantum computing and quantum state manipulation.

## Results and discussions

### Energy matching of subsystems for coupling

First of all, the optical modes in a bare plasmonic lattice without emitters are characterized, and then, they are regulated to interact with the exciton of quantum emitters to reach the strong coupling regime. The Al columns with a radius of 50 nm and height of 50 nm are arranged in a square lattice with a period of 400 nm to generate SLR modes.

**Fig. 1:**
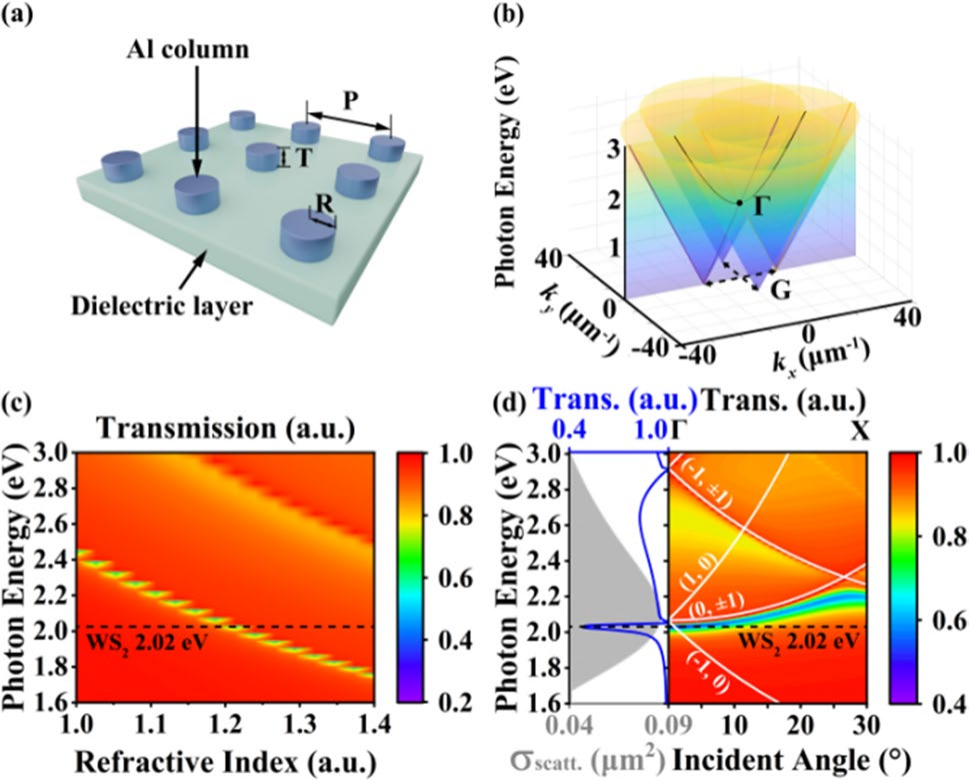
Schematic diagram of the SLR generation process. **a** Al column array structure with *R* = 50 nm, *T* = 50 nm and *P* = 500 nm is placed on the dielectric layer. **b** Light-cone diagram with the angle dependence of the *P* = 500 nm periodic array obtained by the empty-space lattice approximation (ELA) simulation. The black line is the (0, 1)/(0, –1) DO. **c** SLR dispersion diagram as a function of the medium refractive index. **d** Dispersion diagrams of Al plasmonic periodical array derived from angle-dependent transmission spectra along high symmetry excitation conditions after regulation. The gray part (left panel) shows the scattering cross section of an individual Al column. The blue solid line (left panel) is the transmission spectrum of SLR mode under normal incidence that extracts from the right panel. White solid lines represent the DOs and the quantum emitter’s energy is represented as the black dotted line

The SLR caused by the radiative coupling of periodic nanoparticles is generated through regulating the shape and size of the LSPR mode to interact with the DOs. LSPR is manipulated by thickness T, radius R and the ambient refractive index *n*. A typically schematic diagram of the Al periodical nanocolumns array that could generate SLR mode is displayed in Fig. 1a.

A smaller R and T will result in smaller *V*_mode_ and greater field enhancement. Therefore, the radius of the columns is set to 50 nm which is small enough compared with the whole system. DOs are influenced by the period P and the surrounding refractive index *n*, which are geometry parameters from the periodic columns.

Thus, the coupling strength depends on the interdistance between the spectral center wavelength of two modes and the generated SLR closely follows the dispersion of DOs, which can be described by the empty lattice approximation (ELA). The dispersion relation of the light in free space:

1 $$E = \frac{\hbar c}{n}\sqrt {k_{x}^{2} + k_{y}^{2} + k_{z}^{2} } ,$$

where *E* is the photon energy, $\hbar = h/2\pi$ is the reduced Planck constant, *c* is the speed of the light, *n* is the refractive index, *k* is the absolute value of the wave vector and the component in the (*x*, *y*, *z*) axis is *k*_*x*_, *k*_*y*_ and *k*_*z*_, respectively. If *k*_||_ is constant, then *k*_*z*_ is 0, the dispersion relation defines a cone surface in the three-dimensional space known as a light cone, and plane waves can propagate freely inside the cone which is usually called in-plane mode. But outside the light cone, at least one wave number must become imaginary to satisfy the dispersion relation in the above equation, and this is the case for evanescent waves.

Then, the periodical boundary condition should be considered, and a 2D infinite lattice with a periodicity of *p*_*x*_ in the *x*-direction and *p*_*y*_ in the *y*-direction is assumed in this work. The reciprocal lattice vectors are $G_{x} = \pm 2\pi /p_{x} \hat{x}$, $G_{y} = \pm 2\pi /p_{y} \hat{y}$. Here, the refractive index is set as 1.2 and the *p*_*x*_ is as 400 nm. After vector composition, the dispersion relation is expressed as:

2 $$E = \frac{\hbar c}{n}\sqrt {\left( {k_{x} \pm G_{x} } \right)^{2} + \left( {k_{y} \pm G_{y} } \right)^{2} } .$$

The light cone is duplicated and shifted in-plane *x*–*y* and forms the array in Fig. 1b, but the energy of each cone has not been changed as the absolute value of the wave vector is fixed. These light cones are the first-order DOs that will be discussed later.

Depending on the polarization and the propagation direction, the two obvious directions where the light-cone interaction in square lattice could be classified as two modes: transverse electric (TE) mode and transverse magnetic (TM) mode. Under these two different polarization directions, nanoparticles would behave distinctive characters. The (0, 1)/(0, −1) mode excited by the TM is shown in Fig. 1b as the black solid line when *G*_*y*_ = 0 and *k*_*y*_ = 0 in the dispersion. The (1,0)/(− 1,0) diffraction mode (purple solid line and yellow solid line in Fig. 1b) excited by the TE mode is observed after *k*_*x*_ = 0 and *G*_*y*_ = 0. The crossing point of the diffraction is significant as that is the degenerate point Γ where the propagation mode opens a photon band gap and the degenerate DO mode divides into two single DOs. In addition, this degenerate point has a higher density of states than the others, which is conducive to strengthening the light–matter interaction.

After determining the incidence angle, as mentioned above, the refractive index n of different dielectric layers will have an impact on the energy matching of SLR mode and the quantum emitter. When the structural parameters are fixed, the refractive index n is changed from 1.0 to 1.4. The transmission spectra reveal the dispersion of the SLR mode in Fig. 1c. As the refractive index increases, the photon energy of the SLR mode gradually redshifts from 2.4 to 1.78 eV. The refractive index *n* = 1.2 is chosen to match the energy of the quantum emitter. This also shows the refractive index is one of the important freedom degrees to regulate the SLR mode.

Besides, there are also some other free degrees in regulating, such as the radius, height of the Al columns and incident angle for further using a real dielectric material practically.

Figure 1d shows after regulation that a SLR mode with a central energy of 2.01 eV is generated. The right panel of Fig. 1d is the angular resolution dispersion under TM polarization excitation. It is also the crossing section along the Γ-X direction as shown in Fig. 1b. DOs are depicted as the white solid lines. The energy at the Γ point is 2.01 eV which matches well with the peak of the LSPR. When a single metal column is constructed, only a faint LSPR mode with a scatting intensity of 0.083 $\upmu {\text{m}}^{2}$ can be observed in the left panel of Fig. 1d. After the array is arranged and regulated, a narrow line width, asymmetric peak shape’s SLR mode appears (blue line) due to the interaction of the DO with the LSPR. The quality factor $Q$ of the SLR rises 66 and its FWHM is suppressed to 38 nm. The adjusted SLR matches the energy peak of WS_2_ to satisfy the strong coupling generation condition.

### Strong coupling between SLRs and excitons

**Fig. 2:**
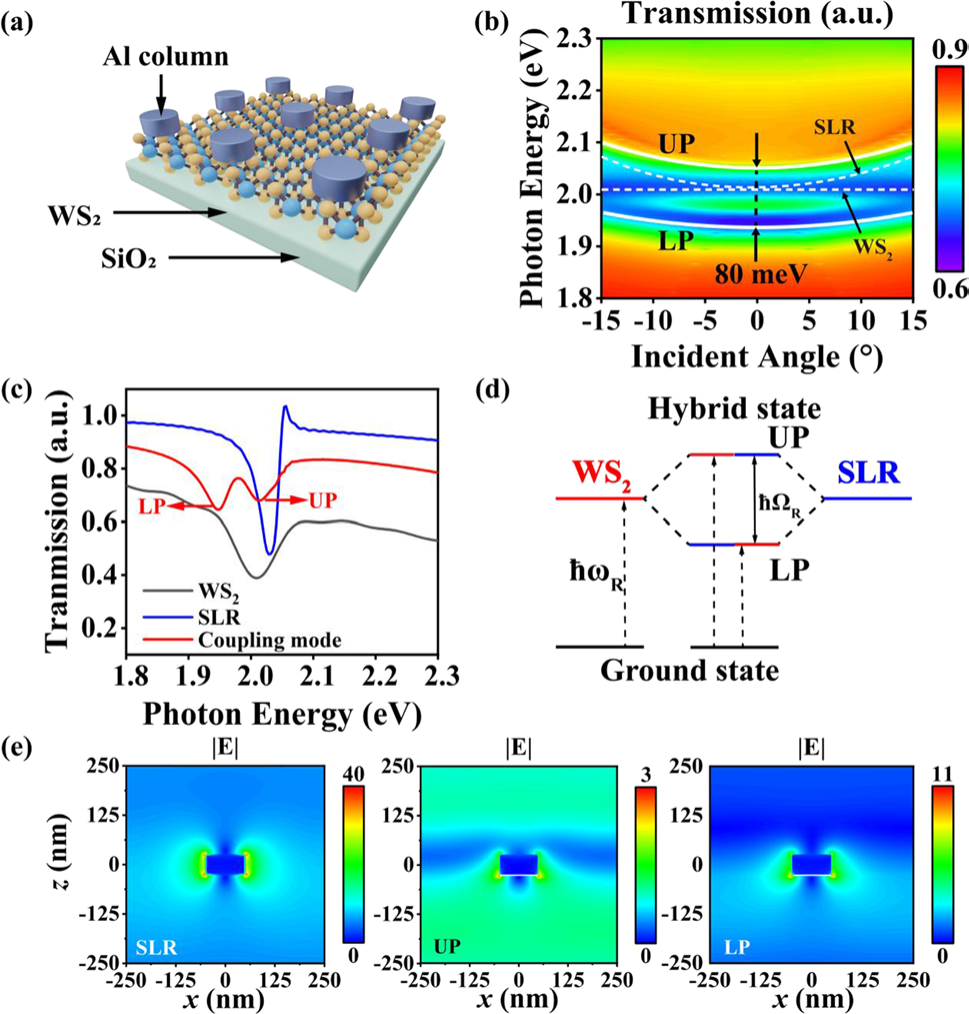
Strong coupling at the Γ point (*k*_||_= 0) of the surface Brillouin zone. **a** Structure schematic of strong coupling in the plasmonic array. **b** Angle-resolved dispersion spectrum of simulated Al column periodic array and quantum emitter strong coupling. The white solid line represents upper polariton/lower polariton, the white dotted line represents the SLR mode and WS_2_ quantum emitter mode, and coupling strength is marked in the diagram. **c** Transmission spectrum of the plasmonic array in-plane wave numbers (*k*_||_= 0). An anti-crossing splitting occurs within a small range of in plane at zero detuning. **d** Schematic diagram of resonance level of two-level system and the cavity mode. **e** Steady-state electric field of SLR and UP/LP at 0° incident angle before and after strong coupling

A monolayer of WS_2_ is added between the metal column array and the dielectric layer to offer excitons in this system as presented in Fig. 2a.

For the definition of strong coupling, two common conditions are used: $2\left| g \right| > \left| {\frac{{\gamma_{{{\text{cav}}}} - \gamma_{{{\text{ex}}}} }}{2}} \right|$ and $\Omega > \frac{{\gamma_{{{\text{cav}}}} }}{2} + \frac{{\gamma_{{{\text{ex}}}} }}{2}$, $\gamma_{{{\text{cav}}}}$ and $\gamma_{{{\text{ex}}}}$ are the damping of the two states, $g$ is the coupling strength and $\Omega$ is the Rabi splitting. The first condition guarantees the generation of strong coupling. If the first condition is satisfied, the system is in strong regime. But the two spectral peaks may not be observed. Only when these two conditions are satisfied simultaneously, the strong coupling can be spectrally observed.

When the transmission peak of WS_2_ overlaps the energy of the SLR pattern, an anti-crossing of the two modes is observed. This could be considered as a mode splitting and strong coupling of the SLR with the quantum emitter. In this paper, $g$ is 40 meV, and the $\gamma_{{{\text{cav}}}}$ and $\gamma_{{{\text{ex}}}}$ are 0.05 eV and 0.065 eV which satisfies both conditions. Besides, the noticeable Rabi splitting in Fig. 2c also proves the generation of strong coupling.

The coupling constant obtains from the dispersion of the simulation is $\hbar g = 40\,{\text{meV}}$ as shown in Fig. 2b. New exciton–polariton modes with half-light and half-matter are produced. White solid lines denote the upper polariton (UP) and lower polariton (LP) branches after splitting.

The spectral features of the newly formed polariton modes at 0° are extracted to analyze the optical response of the coupled system as shown in Fig. 2c, which facilitates a better understanding of the plasmon–exciton coupling features. The noninteracting spectra of the WS_2_ and SLR modes are indicated by black and blue solid lines, respectively. The splitting between the UP and LP is depicted using the red line, further demonstrating the generation of strong coupling.

The transmission intensity of the UP and the LP is reduced compared to the two subsystems without the interaction. This could be explained by the dissipation of WS_2_ quantum emitter. Though the interaction increases, the intrinsic loss of WS_2_ still cannot be ignored. The theoretical energy level diagram of the strong coupling system is illustrated in Fig. 2d, where the exciton can be reduced to a two-level system with a ground-state transition energy of $\hbar \omega_{R}$. When the interaction enters the strong coupling regime, a coherent energy transfer occurs between the two-level system and the plasmon mode. The principle of energy level splitting is as follows. Generally, if the two degenerate energy levels of SLR and exciton overlapping at the frequency spectrum, respectively, then a revisable pathway of energy exchanging between SLR and exciton exists, the states can beat over the two energy levels with an oscillating frequency of $\Omega_{R}$, and then, the final states are the oscillating states with oscillating frequencies of $\omega_{0} \pm \Omega_{R}$, which results in an energy level splitting of $2\Omega_{R}$. In other words, an energy level splitting of $2\Omega_{R}$ forms [27].

The coherent coupling energy of UP and LP is $\hbar \Omega_{R}$ = 80 meV. Therefore, the Rabi splitting of this system is $\hbar g$ = 40 meV. The hybrid states UP and LP, namely the entangled states, can be elaborated later using the full quantum model.

The spatially electric field distributions of samples before and after strong coupling are shown in Fig. 2e. The electric field of SLR is highly concentrated on the nanogap. However, the electric field of the UP and LP modes is mainly concentrated on the bottom of the nanogap. This indicates that the interaction occurs at the intersection surface of the Al column and the WS_2_, and the electric dipole mode mainly participates in the interaction. The maximum intensities of UP (3) and LP (11) are slightly weaker than that of the SLR pattern (Fig. 2c). This is because the spectral transmission of UP and LP is weakened, resulting in an enhanced absorption effect.

### Full quantum model under Heisenberg picture

To deeply dig into the physics underlying the purposed strong coupling system, a quantum model based on the Heisenberg picture is established and calculated by Quantum Toolbox in Python (QuTiP). By using the Jaynes–Cummings model, the mode can be used to calculate the time evolution of a quantum state and for a quantized bosonic field interacting with a two-level atom. And here the excitonic mode is quantized as a two-level atom by the two-level approximate approach. Herein, the SLR mode and excitonic mode are quantized as bosonic mode and fermionic mode.

Considering the electromagnetic field within a one-dimensional confined cavity of length $L$ and assuming the electric field is x-polarized and transmits along *z*-axis, the electric field component can be expressed as ${\varvec{E}} = {\varvec{e}}_{x} E_{x} \left( {z,t} \right)$,and it can be expanded as standing wave form:

3 $${\varvec{E}} = {\varvec{e}}_{x} E_{x} \left( {z,t} \right) = {\varvec{e}}_{x} \mathop \sum \limits_{n} A_{n} q_{n} \left( t \right)\sin k_{n} z = \mathop \sum \limits_{n} E_{n} ,$$

where $k_{n} L = n\pi ,n \in {\mathbb{N}}$, and $k_{n}$ is the wave vector of the *n*th standing wave mode.

According the Maxwell equations, the magnetic field is y-polarized and is expressed as:

4 $${\varvec{B}} = {\varvec{e}}_{y} B_{y} \left( {z,t} \right) = {\varvec{e}}_{y} \mathop \sum \limits_{n} \frac{{A_{n} }}{{\omega_{n} c}}p_{n} \left( t \right)\cos k_{n} z = \mathop \sum \limits_{n} B_{n} ,$$

where $$\omega_{n} = ck_{n}$$ and $$p_{n} \left( t \right) = \dot{q}_{n} \left( t \right)$$.

The energy of electromagnetic field is $H = \frac{1}{2}\int {{\text{d}}V\left( {\varepsilon_{0} {\varvec{E}}^{2} + \frac{1}{{\mu_{0} }}{\varvec{B}}^{2} } \right)}$; substituting Eqs. (3) and (4) into the expression, we get:

5 $$H = \mathop \sum \limits_{n} q_{n}^{2} \left( t \right) + \frac{{p_{n}^{2} \left( t \right)}}{{\omega_{n}^{2} }} = \mathop \sum \limits_{n} H_{n} ,$$

where $H_{n}$ is the energy of the nth standing wave mode and $\frac{{A_{n}^{2} L^{2} }}{8}\varepsilon_{0} = 1$. Here, $p_{n}$ and $q_{n}$ are set as operators and obey the commutation relations as follows:

6 $$\left[ {q_{j} ,p_{k} } \right] = i\hbar \delta_{jk} ,\quad \left[ {q_{j} ,q_{k} } \right] = 0,\quad \left[ {q_{j} ,q_{k} } \right] = 0.$$

Next, the following operators are introduced:

7 $$\hat{a}_{n} = \sqrt {\frac{1}{{\hbar \omega_{n} }}} \left( {q_{n} + i\frac{{p_{n} }}{{\omega_{n} }}} \right),$$

8 $$\hat{a}^{\dag }_{n} = \sqrt {\frac{1}{{\hbar \omega_{n} }}} \left( {q_{n} - i\frac{{p_{n} }}{{\omega_{n} }}} \right),$$

where the following commutation relations are satisfied:

9 $$\left[ {\hat{a}_{j} ,\hat{a}^{\dag }_{k} } \right] = \delta_{jk} ,{ }\left[ {\hat{a}_{j} ,\hat{a}_{k} } \right] = 0,{ }\left[ {\hat{a}^{\dag }_{j} ,\hat{a}^{\dag }_{k} } \right] = 0.$$

Substituting Eqs. (7) and (8) into Eq. (5), we get:

10 $$H = \mathop \sum \limits_{n} H_{n} = \mathop \sum \limits_{n} \hbar \omega_{n} \hat{a}_{n}^{\dag } \hat{a}_{n} .$$

The matter–classical field interaction and the matter–quantum field interaction can be understood in a consistent way as follows: In the both cases, the energy oscillates periodically between its ground state and excited state. In the case of matter–classical field interaction, only the matter is quantized; therefore, the phenomena related to the quantization of the field cannot be explained well, such as the spontaneous emission. And this phenomenon can be explained well in the view of matter–quantum field interaction.

The total Hamiltonian *H* of the established quantum mechanical model includes the following terms:

The first term is the system Hamiltonian,

11 $$H_{{{\text{sys}}}} = \hbar \omega_{1} \hat{a}^{\dag } \hat{a} + \frac{{\hbar \omega_{2} }}{2}\hat{\sigma }_{z} + \hbar g\left( {\hat{a}^{\dag } \hat{\sigma }_{ - } + \hat{a}\hat{\sigma }_{ + } } \right),$$

where $H_{{\text{F}}} = \hbar \omega_{1} \hat{a}^{\dag } \hat{a} + \frac{{\hbar \omega_{2} }}{2}\hat{\sigma }_{z}$ is the free-particle Hamiltonian of the two subsystems, $\omega_{1}$ and $\omega_{2}$ denote the resonance frequencies of SLR and excitonic modes, $\hat{a}^{\dag }$ and $\hat{a}$ are the creation and annihilation of SLR mode, $\hat{\sigma }_{z}$ is the Pauli operator of excitonic mode, and $\hat{\sigma }_{ - }$ and $\hat{\sigma }_{ + }$ are the lowering and raising operators of the excitonic mode, respectively, with $\hat{\sigma }_{z} = \left[ {\hat{\sigma }_{ + } ,\hat{\sigma }_{ - } } \right]$. $H_{I} = \hbar g\left( {\hat{a}^{\dag } \hat{\sigma }_{ - } + \hat{a}\hat{\sigma }_{ + } } \right)$ is the Hamiltonian of the coupling between SLR mode and excitonic mode with a coupling strength g under the rotating-wave approximation (RWA).

The second term is the reservoir Hamiltonian,

12 $$H_{{\text{R}}} = \mathop \smallint \limits_{ - \infty }^{ + \infty } \left[ {\hbar \omega^{\prime}\hat{b}^{\dag } \left( {\omega^{\prime}} \right)\hat{b}\left( {\omega^{\prime}} \right) + \hbar \omega^{\prime}\hat{c}^{\dag } \left( {\omega^{\prime}} \right)\hat{c}\left( {\omega^{\prime}} \right)} \right]{\text{d}}\omega^{\prime}$$

The reservoir follows the Bose–Einstein distribution at thermal equilibrium, where $\hat{b}^{\dag }$ ($\hat{b}$) and $\hat{c}^{\dag }$ ($\hat{c}$) are the creation (annihilation) operators of the reservoir that interact with SLR mode and excitonic mode, respectively.

The next term that describes the interactions between the coupling system and the reservoir is the radiative and nonradiative damping of the subsystems:

13 $$H_{{{\text{SR}}}} = i\hbar \mathop \smallint \limits_{ - \infty }^{ + \infty } \sqrt {\frac{{\gamma_{1} }}{2\pi }} \left[ {\hat{b}^{\dag } \left( \omega \right)\hat{a}\left( \omega \right) - \hat{a}^{\dag } \left( \omega \right)\hat{b}\left( \omega \right)} \right] + \sqrt {\frac{{\gamma_{2} }}{2\pi }} \left[ {\hat{c}^{\dag } \left( \omega \right)\hat{\sigma }_{ - } \left( \omega \right) - \hat{\sigma }_{ + } \left( \omega \right)\hat{c}\left( \omega \right)} \right]{\text{d}}\omega ,$$

where $\gamma_{1}$ and $\gamma_{2}$ are the damping rate of SLR mode and excitonic mode.

The last term is the driven field Hamiltonian that describes the driven field with a Gauss wave packet:

14 $$H_{d} = 2\hbar ge^{ - i\omega t} e^{{ - (t/\tau )^{2} }} \left( {\hat{a} + \hat{\sigma }_{ - } } \right) + h.c.,$$

where $\omega$ and $\tau$ are the frequency and pulse width of the driven field, respectively. Therefore, the driven field is the essential component to have a high degree of entanglement states.

The total Hamiltonian is $H = H_{{{\text{sys}}}} + H_{{\text{R}}} + H_{{{\text{SR}}}} + H_{{\text{d}}} .$ Under the Heisenberg picture, the total Hamiltonian follows the Heisenberg equations of motion: $\dot{\hat{O}} = \left[ {\hat{O},H} \right]/\left( {i\hbar } \right)$ for any operator $\hat{O}$. Therefore, the photon numbers, $\hat{a}^{\dag } \hat{a}$ and $\hat{\sigma }_{ + } \hat{\sigma }_{ - }$, which describe the time evolutions of subsystems in the strong coupling system, can be calculated using the Heisenberg equations of motion giving the initial condition $\hat{a}^{\dag } \hat{a} = 0$ and $\hat{\sigma }_{ + } \hat{\sigma }_{ - } = 0$. By diagonalizing the system Hamiltonian $H_{{{\text{sys}}}}$, the eigenfrequencies of the strong coupling system are obtained as follows:

15 $$\Omega_{ \pm } = \frac{{\omega_{1} + \omega_{2} \pm \sqrt {4g^{2} + \Delta^{2} } }}{2},$$

where $\Delta = \omega_{1} - \omega_{2}$ is the detuning between two subsystems.

**Fig. 3:**
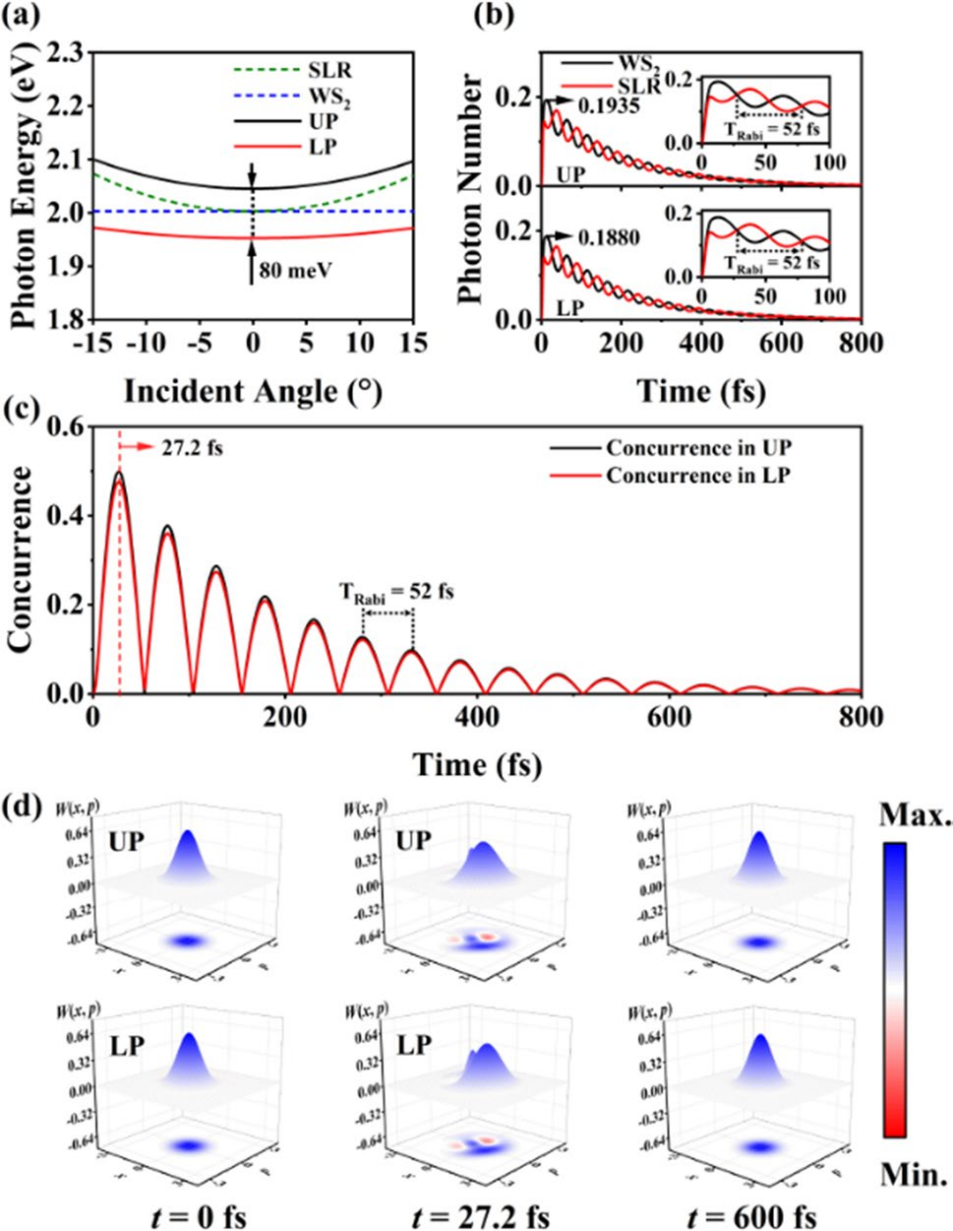
Quantum model calculation of the strong coupling system. **a** Eigenfrequency calculations of UP and LP modes. **b** Time evolution of average photon numbers of two subsystems (SLR mode and WS_2_ mode) within the UP and LP modes within the strong coupling system at normal incidence. **c** Time evolution of concurrences between two subsystems within the UP and LP modes, respectively. **d** Wigner functions of UP and LP modes at *t* = 0, 27.2 fs and 600 fs

Figure 3a displays the result for the photon energy of the UP and LP modes as a function of incident angle. The anti-crossing splitting is obtained with the reflection spectra as a function of incident angle, indicating the strong coupling between SLR mode and excitonic mode. The time evolution of the average photon number of SLR mode and excitonic mode in UP and LP modes is illustrated in Fig. 3b. In each polaritonic mode, SLR mode and excitonic mode periodically exchange photon numbers with a Rabi period $T_{{{\text{Rabi}}}} = 52\,{\text{fs}}$ and decay simultaneously due to radiative and nonradiative loss of two subsystems.

With the purpose of analyzing the photon entanglement induced by the strong coupling, the concurrence is employed to justify entanglement states between two subsystems [28], as illustrated in Fig. 3c. Initially, the concurrence in each polaritonic mode starts increasing from 0 with unentangled initial states, as a symbol of forming the entangled states, followed by the manifestation of Rabi oscillation with a Rabi period $T_{{{\text{Rabi}}}} = 52\,{\text{fs}}$ and simultaneous decay as a result of the system’s loss.

The highest concurrence has reaches to 0.5 at 27.2 fs which is a big boost. Though the concurrence from the plasmonic lattice exhibits a fast decay process about 600 fs, the study of this ultrafast dynamics of physical processes could still be measured by applying the fs transient absorption spectroscopy technology.

To get further valuable insight into the nature of the quantum states of UP and LP modes within the strong coupling system, the Wigner functions of the corresponding modes are also investigated under the quantum mechanical model. The Wigner function is the quasi-probability distribution function defined in the phase space of (*x*, *p*), which is real-valued and dimensionless. It is equivalent to the wave function or density matrix when describing the quantum states; besides, it contains all the information of quantum states and can be described by the following formula:

16 $$W\left( {x,p} \right) = \frac{1}{\pi }\mathop \smallint \limits_{ - \infty }^{ + \infty } \exp (ipy)\left. {x - \frac{y}{2}} \right|\hat{\rho }\left| {x + \frac{y}{2}} \right.{\text{d}}y,$$

where the density matrix of the modes within the coupling system is defined as $\hat{\rho } = \sum\nolimits_{\varphi } {P_{\varphi } \left| \varphi \right.\left. \varphi \right|}$; it represents the probability of the system in the state $\left| \varphi \right.$.

As illustrated in Fig. 3d, the initial states of the UP and LP modes can be considered as vacuum states at *t* = 0 fs, and there is no entanglement states existing in the two subsystems. At *t* = 27.2 fs, the concurrences reach to the maximum in Fig. 3c and the Wigner functions for both modes present negative components, which indicates they are nonclassical state (entangled state) at this moment. During a long decoherence process (600 fs), the nonclassical state is very weak and the concurrence trends to zero as vacuum state.

## Conclusion

We proposed a composite structure consisting of a periodic nanoarray and a quantum emitter to achieve strong coupling between SLR and excitons. In this work, the strong coupling occurred at the degenerate point, due to the large density of states at the degenerate point and the strong local electric field characteristics of the SLR mode. It greatly promoted the coupling between the SLR mode and the exciton, and realized the Rabi splitting of $\hbar {\text{g}}$ = 40 meV. The interaction between SLR and excitons was characterized in angle-resolved dispersion spectra, which provided more degrees of freedom to manipulate strong coupling. In addition, a full quantum model was proposed to fit the strong coupling phenomenon. The results showed that when the detuning of two subsystems was 0, concurrence, the quantized entanglement degree, was measured, and the maximum concurrence was 0.5. Finally, the nonlocal coherence properties of entangled states were analyzed. This provided a direction for manipulating the strong coupling system to regulate entanglement. It provided the possibility of exploring entanglement generation through strong coupling between SLR and excitons which allows us to adjust the nonlocality and correlation between quantum states. This whole quantum method to analyze entanglement constitutes a key step toward a new form of efficient, all-optical and quantum information processing.

## Data Availability

The datasets supporting the conclusions of this article are included within the article.
